# Towards a Better Understanding of Texturization during High-Moisture Extrusion (HME)—Part II: Characterization of Thermophysical Properties of High-Moisture Meat Analogues

**DOI:** 10.3390/foods12122283

**Published:** 2023-06-06

**Authors:** Elisabeth Högg, Cornelia Rauh

**Affiliations:** Department of Food Biotechnology and Food Process Engineering, Technische Universität Berlin, 14195 Berlin, Germany

**Keywords:** data-driven prediction models, high-moisture extrusion, high-moisture meat analogues (HMMAs), thermophysical properties of HMMAs, protein texturization

## Abstract

It is crucial to determine the thermophysical properties of high-moisture extruded samples (HMESs) to properly understand the texturization process of high-moisture extrusion (HME), especially when the primary objective is the production of high-moisture meat analogues (HMMAs). Therefore, the study’s aim was to determine thermophysical properties of high-moisture extruded samples made from soy protein concentrate (SPC ALPHA^®^ 8 IP). Thermophysical properties such as the specific heat capacity and the apparent density were experimentally determined and further investigated to obtain simple prediction models. These models were compared to non-HME-based literature models, which were derived from high-moisture foods, such as soy-based and meat products (including fish). Furthermore, thermal conductivity and thermal diffusivity were calculated based on generic equations and literature models and showed a significant mutual influence. The combination of the experimental data and the applied simple prediction models resulted in a satisfying mathematical description of the thermophysical properties of the HME samples. The application of data-driven thermophysical property models could contribute to understanding the texturization effect during HME. Further, the gained knowledge could be applied for further understanding in related research, e.g., with numerical simulation studies of the HME process.

## 1. Introduction

Thermophysical properties of feed, agricultural, and food products are often required for process engineering design of apparatus and operation or optimization of processes. Data on thermophysical properties such as enthalpy (H), specific heat capacity (c_p_), apparent density (ρ), thermal conductivity (k), and thermal diffusivity (α) are required to predict heat transfer rates in foods, especially when designing heating, cooling, or freezing processes [1,2]. Further information on the definition of these thermophysical properties can be found elsewhere [3,4,5,6,7,8,9,10].

One of the most significant limitations in designing and optimizing thermal food processing systems is the lack of data on thermophysical properties of food [11]. To overcome the limitation of experimental data on thermophysical properties, mathematical models have been developed to predict these for all conditions and compositions of foods. Some of these models are based on the properties of their food-related components (e.g., fats, carbohydrates, proteins, etc.), since the thermophysical properties of foods are strongly dependent on chemical composition and temperature, whereas other models are directly linked to a certain type of food, e.g., soy dough, tofu, meat, and fish products [4,11,12,13,14,15,16,17,18,19]. Due to the dependence on the chemical composition, thermophysical properties of foods can be predicted. Nevertheless, experimental measurements are the primary source for thermophysical modeling, and a comprehensive literature search revealed limited experimental data on thermophysical properties of plant-based proteins with a high moisture content (≥50%) and there are no existing data for high-moisture extruded samples. For some thermophysical properties, the analyses can be quite direct, whereas other analyses may be more challenging. For example, the apparent density of solid foods can be determined via a simple geometric dimension method, whereas the specific heat capacity of a food system can be measured via a sensitive differential scanning chromatography (DSC) analysis approach or estimated via mathematical models as the sum of the specific heat capacities of its individual components, as it is an additive property and not food structure dependent [20,21]. In general, for calculating thermal food processing systems, the specific heat capacity is an important consideration. The specific heat capacity of food materials shows a temperature dependency and, thus, changes with temperature [22].

Thermal diffusivity is dependent on heat capacity, density, water content, and thermal conductivity. Thermal conductivity is a structure-dependent property and depends on fiber or porous structure. This dependency makes it more challenging to model. However, models based on the electrical resistance analogy by considering the direction of heat flow through the food can be applied [23].

High-moisture extrusion (HME) is a multistep thermomechanical process, which involves the combination of heating, mixing, shearing, kneading, and subsequent cooling of a protein–water mixture to facilitate texturization of multilayered fibrous structures with a meat-like texture [24,25,26]. Therefore, data on thermophysical properties are important to describe the texturization effects of a protein–water mixture throughout the HME process. To gain insights into the texturization mechanisms of plant-based proteins, the knowledge of their thermophysical properties is a prerequisite for evaluating texturization effects during HME. All thermomechanical processes during HME (e.g., in the screw and cooling die section) are of great importance, as their interaction is responsible for the entire outcome of the HME process in terms of product-related attributes. Currently, there is no literature source available describing the use of thermophysical properties to explain the texturization effect during HME processing. Thus, the aim of this work was to determine the above-mentioned thermophysical properties of high-moisture extruded samples made from soy protein concentrate to shed light into the black box of HME texturization. The specific heat capacity and apparent density were obtained experimentally and further researched to develop simple prediction models. The experimentally measured thermophysical values were compared with predicted values based on mathematical equations from the literature, which are related to food but not directly to high-moisture extruded samples [12,13,20,23,27,28]. Further, the measured values of the specific heat capacity and apparent density were used to predict and compare thermal conductivity and thermal diffusivity using mathematical model equations based on the literature [12,13,20,23,27,28]. The models were selected to calculate thermophysical properties of high-moisture extruded samples (HME samples), because the models were based: (i) on food constituents and thus cover a broad range of food products, (ii) on soy protein ingredients, or (iii) on meat or fish products, which should be mimicked by HME.

## 2. Material and Methods

### 2.1. Raw Materials

A soy protein concentrate (SPC ALPHA^®^ 8 IP) was purchased from Solae Europe S.A. (Solae LLC, St. Louis, MO, USA) to determine thermophysical properties of its high-moisture extruded products. The composition of the plant-based raw material, which is often used as a benchmark reference ingredient for HME by the food industry, is listed in Table 1 based on the supplier’s specification. In further discussion, SPC ALPHA^®^ 8 IP will be referred to as SPC Alpha 8.

### 2.2. High-Moisture Extrusion (HME)

SPC Alpha 8 was textured by HME using a laboratory, co-rotating twin screw extruder ZSK 25 from Werner & Pfleiderer (Coperion GmbH, Stuttgart, Germany) with an outer screw diameter of Da = 25 mm and a length–outer diameter ratio of L/Da = 28.8. The material temperature (T_in_) and extruder pressure (p_in_) were measured directly before the cooling die inlet, and the specific mechanical energy (SME) was calculated based on the equation developed by Meuser and van Lengerich [29]. High-moisture extruded samples at three different water contents were available from previous trials, and details on the experimental setup can be found in Högg and Rauh [30]. Based on the experimental design of this study and its results, five high-moisture extruded samples at different water contents and with defined textural attributes (well-textured (HMMA), textured, and poorly textured) were selected (Table 2), in order to study the influence of water content and/or texture on thermophysical properties. Sample No. 3 in Table 2 was originally not part of the experimental design from Högg and Rauh [30]. This sample was included to allow a comparison of three different textures at the same water content of 60%. 

The thermophysical properties such as specific heat capacity, apparent density, thermal diffusivity, and thermal conductivity of the samples listed in Table 2 were determined. The methods and/or mathematical models used are described in detail below.

The sensory evaluation and clustering of the HME samples were performed as described by Högg and Rauh [30]. The HME samples were clustered into well-textured, textured, and poorly textured based on the following criteria (Table 3):

### 2.3. Determination of Apparent Density

The apparent density was measured by the geometric dimension method, which is a simple and accurate method for regularly shaped foods [31,32,33,34,35] At least 10 regularly shaped specimens were punched out of one HME sample (Table 2) using a 0.016 m diameter core cutter. Each specimen was weighed using a laboratory scale (±0.0001 g, Scaltec SBA 31, Scaltec Instruments GmbH, Göttingen, Germany) and its characteristic dimension was measured using a caliper (±0.01 mm, Globaltronics GmbH & Co. KG, Hamburg, Germany). Weight and dimensions were converted into kg and m, respectively, and the apparent density of each HME sample was calculated as an average of the individual measurements (n = 10) using Equation (1):

(1) $$\rho_{ap}=\frac{1}{n}\sum_{i=1}^{n}\frac{m_{i}}{V_{i}}=\frac{1}{n}\overset{n}{\underset{i=1}{\sum }}\frac{m_{i}}{\pi \;r_{i}{}^{2}h_{i}}$$

where *ρ_ap_* is the apparent density (kg/m^3^), m is the weight of the cylindrical sample (kg), *r* is the radius of the cylindrical sample (m), and *h* is the height of the cylindrical sample (m).

Preliminary tests were performed to study the influence of texturization degree on the apparent density. Therefore, high-moisture extruded samples that depicted a well-defined, defined, and non-multilayered fibrous structure and were extruded with a water content of 60% were selected and analyzed (Table 2, sample No. 1, sample No. 2, and sample No. 3). Subsequently, prediction models were derived from the calculated density values. In addition, the model obtained in this study was compared with models from the literature (more information see Last Table, page 13–14) [4,20,28,36].

### 2.4. Determination of Specific Heat Capacity by µDSC

The calorimetric measurements were performed using a microcalorimeter (µDSC7 EVO, SETARAM Instrumentation, Caluire, France) in a temperature range of 40 °C to 115 °C with a heating rate of 0.2 K min^−1^. The sample cell of the µDSC was filled with 0.6500 g of sample and accurately weighed to the nearest 0.0001 g. The initial temperature of 40 °C was chosen due to the cooling die temperature, which was set to 40 °C.

Before the HME samples were analyzed, a blank test was performed with an empty cell, and the specific heat capacity c_p_ of distilled water was determined. The uncertainty of the analysis was derived by comparing the measured c_p_ of water with the c_p_ of water found in the literature and all further measured data were corrected with an uncertainty of 5.6°% as described by Yu and Christie [37] and Zhang et al. [38].

In addition to the blank test and the c_p_ determination of distilled water, preliminary tests were performed to investigate the influence of (1) the cutting procedure and geometrical shapes as well as (2) the texturization degree on the determination of specific heat capacity, as extrusion conditions can influence thermophysical properties [39]. Therefore, HME samples that were well-textured, textured, and poorly textured with a water content of 60% (*w*/*w*, on a dry basis) were selected and calorimetrically analyzed (Table 2, sample No. 1, sample No. 2, and sample No. 3). The samples were either cut into slices (1 mm × 5 mm × 19 mm, H × W × L) or cubes (1 mm × 1 mm × 1 mm, H × W × L).

To evaluate the influence of a possible denaturation reaction on the specific heat capacity of HMESs, a rescan was performed in which an initial DSC scan was performed, then the sample was cooled to 40 °C within 1.5 h, equilibrated at 40 °C for 1 h, and re-scanned.

Based on the results of the preliminary tests, further calorimetric analyses were carried out with HME samples extruded at water contents of 65 and 70%.

The mass loss due to water evaporation during measurements was <0.09%. The specific heat capacity c_p_ of each sample was calculated as an average of two individual measurements in the temperature range of 40–115 °C and the data were fitted via a linear regression model over the tested temperature range to mathematically describe c_p_. All data were processed with CALISTO software (Version v1.097, SETARAM Instrumentation, Caluire, France).

In addition, the derived models of the samples with different water contents were summarized using a multiple linear regression (MLR) approach as a function of water content and temperature. This MLR model was compared with literature models that studied thermophysical properties based on the composition of the food [4,20], soy-based products such as soy flour, soy dough, and tofu [13,27,28,40], or a meat product [18].

### 2.5. Model Prediction for Thermal Diffusivity

Thermal diffusivity α was predicted using theoretical and empirical mathematical model equations from the literature, including: (1) the model of Choi and Okos [20], which is one of the most common models for predicting thermal diffusivity and which is based on the composition of food products; (2) the prediction model of Wallapapan et al. [13] used for studying the thermal diffusivity of defatted soy flour over a moisture range of 9–39% and densities of 95–1300 kg/m^3^ at a temperature of 130 °C; (3) the model of Wagner [27] used for studying the thermal diffusivity of defatted soy dough in a temperature range of 70–105 °C at moisture levels of 0, 25, and 50%, although only average values over the measured temperature range were available; (4) the prediction model for processed soy protein, or more precisely, the regression model of Baik and Mittal [28] for tofu in a temperature range of 6–74.2 °C and moisture content range of 0.3–0.7 (wb); and (5) the mathematical model from Sweat [12] for meat and fish products (Table 4).

### 2.6. Model Prediction for Thermal Conductivity

The thermal conductivity k was calculated using Equation (2), in which the specific heat capacity (*c_p_*) model and the apparent density (*ρ*) model developed in this work and the thermal diffusivity (*α*, Table 4) derived from the models of Choi & Okos [20] as well as Baik and Mittal [28] were added.

(2) $$k=\alpha \;\rho \;c_{p}$$

In addition to Equation (2), thermal conductivity was also calculated using a model based on the analogy with electrical resistance. This approach takes the structural dependence of thermal conductivity into account. The parallel and series (or perpendicular) thermal conductivity models of Murakami and Okos [23] for multicomponent systems were used.

The parallel model is the sum of the thermal conductivities of the different ingredients of a food multiplied by their volume fractions (Equations (3) and (4)):

(3) $$k_{\|}=\overset{n}{\underset{i=1}{\sum }}E_{i}\;k_{i}$$

with

(4) $$E_{i}=\frac{\frac{M_{i}}{\rho_{i}}}{\sum_{i=1}^{n}\frac{M_{i}}{\rho_{i}}}$$

where *k*_II_ is the thermal conductivity for parallel heat flow, *E_i_* is the volume fraction of the food component, and *k_i_* is the heat conductivity of the food component. *E_i_* can be calculated with Equation (4) where *M_i_* is the mass fraction and *ρ_i_* is the density of the food ingredient.

The series or perpendicular model is the reciprocal of the sum of the volume fractions divided by their thermal conductivities (Equation (5)):

(5) $$\frac{1}{k_{⊥}}=\overset{n}{\underset{i=1}{\sum }}\frac{E_{i}}{k_{i}}$$

where *E_i_* is the volume fraction of the food component and *k_i_* is the heat conductivity of the food component.

A summary of all models applied to calculate the thermal conductivity is shown in Table 5.

### 2.7. Statistical Analysis

SigmaPlot software (SigmaPlot 12.5, Systat Software Inc., San Jose, CA, USA) was used to develop regression models for specific heat capacity and apparent density of the HME samples. To select the best-fitting model, two criteria were considered, the correlation coefficient R^2^ and the F-value of the model. To evaluate significant differences between the c_p_ of different HME samples, the method described by Paternoster et al. [41] was selected, which compares the slopes of individual *c_p_* measurements via a statistical *t*-test (*p* < 0.05).

## 3. Results and Discussion

### 3.1. Characterization of Specific Heat Capacity of High-Moisture Extruded Samples

**Figure 1:**
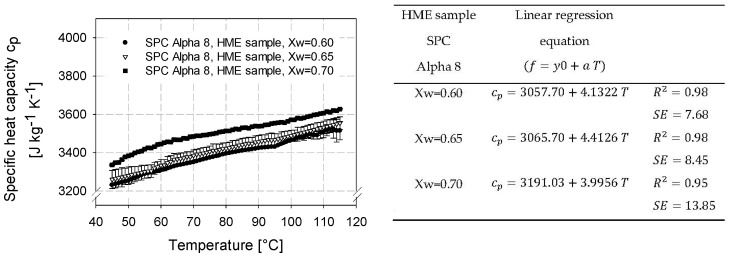
Specific heat capacity for high-moisture extruded samples consisting of soy protein concentrate SPC Alpha 8 as a function of temperature at water mass fractions of Xw = 0.60, Xw = 0.65, and Xw = 0.70.

Based on preliminary tests, where the aim was to study the influence of cutting techniques and texturization degree on the specific heat capacity, the high-moisture extruded samples were cut into slices for further µDSC investigation. This decision to slice the samples relied on the following hypothesis supported by the literature [42,43]. HME samples have a solid structure after coming out of the cooling die, and chopping the HME samples into cubes could increase the surface-to-volume ratio, potentially releasing more surface water and resulting in a falsified and higher c_p_ value than the actual c_p_ value of a solid HME product. Ioannidi et al. [42] and Höhne et al. [43] also found differences in c_p_ measurements due to different cutting techniques and the differences could be explained by temperature distribution. The temperature distribution is more homogeneous in an intact, dense, and solid sample cut into slices than in a compact sample cut into a cube shape. Thus, the homogenous temperature distribution could lead to a more homogeneous heat transfer through the sample. A homogenous temperature distribution and heat transfer are also recommended by DSC instrument manufacturers to generate accurate values. Furthermore, cutting the HME products into slices minimized the effects of mechanical manipulation and kept the HME sample as close as possible to its natural state. Therefore, in order to determine the influence of water on the specific heat capacity, HME samples extruded at water contents of 60, 65, and 70% (*w*/*w*, on dry basis) were analyzed (Table 2). The selected HMESs were evaluated as textured using the sensorial analysis described in Section 2. The results are shown in Figure 1.

The specific heat capacities correlated positively with temperature and, thus, increased with temperature. Furthermore, a dependence of specific heat capacity on water content was evident as well as significant (*p* < 0.05). This was to be expected since water dominates in the HME products [8,27,44,45]. The specific heat capacity increased with increasing water content. For HMESs with different water content, the specific heat capacity increases with increasing water content, as more heat energy is required to raise the temperature of the sample by 1 °C. This is due to the fact that water in the sample can absorb and store more heat energy than other components present, such as fats, fibers, and proteins, which have lower specific heat capacities [46]. For example, the specific heat capacity of water is 4184 J/kg K at 20 °C, while the specific heat capacity of cellulose fibers is 1300–1500 J/kg K [47] and that of proteins (e.g., albumin and globulin) is approx. 1200 J/kg K at 20 °C [48].

Due to the dependence of specific heat capacity on water and temperature, a multiple linear regression ($c_{p}=2341.64+1201.57\;X_{W}+3.8958\;T)$ as a function of mass fraction of water content Xw and temperature T was derived with $R^{2}=0.96$ and SE=18.73. This MLR equation can be applied for predicting specific heat capacity of HME samples made from soy protein concentrate SPC Alpha 8.

The multiple linear regression model with a correlation coefficient of R^2^ = 0.96 can be seen as a good prediction tool for determining specific heat capacities of HME samples made from SPC Alpha 8 in the temperature range between 40 and 115 °C and with water mass fractions of 0.60–0.70 (d.b.).

The mathematical model derived from experimental data was compared with theoretical or empirical methods from the literature, which predict, among others, the specific heat of food products depending on their chemical composition and temperature [4]. Table 6 shows an overview of relevant theoretical or empirical methods from the literature. Models that studied the thermophysical properties of soy-based materials were also selected and compared with our predictive model. Further, a model for predicting the specific heat capacity of turkey meat was included as a comparison with meat products.

When comparing the regression model based on our experimental data with theoretical or empirical models from the literature (Table 6), our experimental data agreed well with the literature models. The percent deviations are listed in Table 6. Unless otherwise noted, the deviation was calculated at a temperature of 90 °C and water content of 60% to compare the literature models with our multilinear regression model.

As shown in Table 6, not all of the used literature models covered the entire parameter domain tested in this study (Table 2). Therefore, to be able to make a comparison with the models of Wallapapan et al. [13], Wagner [27], Baird and Reed [40], and Marcotte et al. [18], the experimentally obtained multilinear regression model as well as the literature models were extrapolated to the valid domain. This was accomplished by retaining one of the parameters of each model (either temperature or water content) in their valid domain. For example, for the comparison of our developed model with the model of Wallapapan et al. [13], a temperature of 130 °C and a water content of 60% were used. Thus, the parameter values that were “outside” of the domains were the mass water fraction of 0.60 for the Wallapapan et al. model and the temperature of 130 °C for our model. A similar approach was followed for all other models.

Using the above-mentioned approach, deviations between the literature models and our developed model could be calculated (Table 6).

Wagner [27] investigated the specific heat capacity of defatted soybean doughs with water contents between 30 and 70% but did not establish an equation for the measured values, so the specific heat could only be compared for MC of 50%. The deviation was −7.23% for a temperature of 90 °C at 50% moisture content.

Baird and Reed [40] published the equation listed in Table 6 based on the results of Heldman and Singh [11]. The equation was valid for c_p_ values at T = 20 °C. Therefore, our MLR model was extrapolated to T = 20 °C and compared to the c_p_ values of Baird and Reed [40]. Percentage deviations of −1.10% indicated good agreement with our MRL model at a moisture content of 60%. Extrapolation of the Baird and Reed [40] model to higher temperatures resulted in higher deviations up to −9% at T = 90 °C. The specific heat of the processed products, such as tofu and turkey, showed a deviation between −1.46 and −5.19% compared to HME products. In particular, the c_p_ values of tofu corresponded very well with our model. Both models were obtained at similar water content and temperature ranges. The two literature models that best describe c_p_ based on the smallest deviation compared to HMESs measured at 90 °C via µDSC are Choi and Okos (0.84%) [4] and Baik and Mittal (1.46%) [28] (Table 6).

### 3.2. Determination of Apparent Density

**Figure 2:**
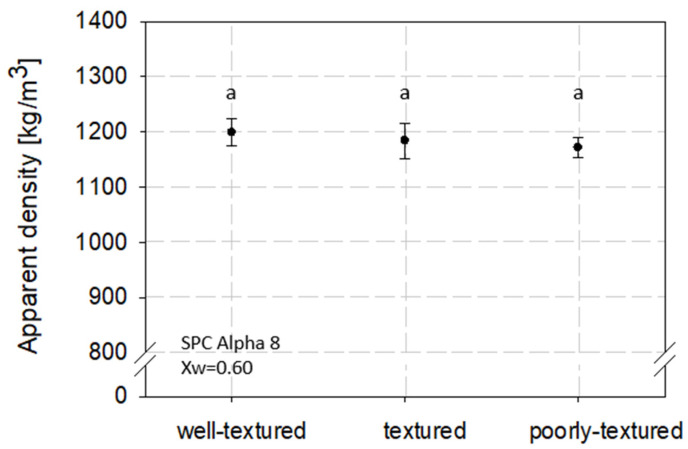
Apparent density of high-moisture extruded samples with different sensory-evaluated textures. Samples were extruded with a moisture content of 60%. Different letters indicate grouping based on significant differences (*p* < 0.05).

The apparent density is defined as the density of the product, including all pores and void spaces [36]. The influence of the texturization degree of high-moisture extruded samples on the apparent density was determined by selecting samples with different textures (Figure 2) but with the same moisture content (Xw = 0.60, Table 2). The apparent density values are plotted versus the subjectively determined texturization, as shown in Figure 2.

The apparent density decreased slightly, but not significantly, with decreasing texturization degree, as shown in Figure 2. A density of 1199.26 kg/m^3^ (±25.15) was determined for a well-textured sample. In comparison, a density of 1172.04 kg/m^3^ (±17.32) was measured for a poorly textured sample.

**Figure 3:**
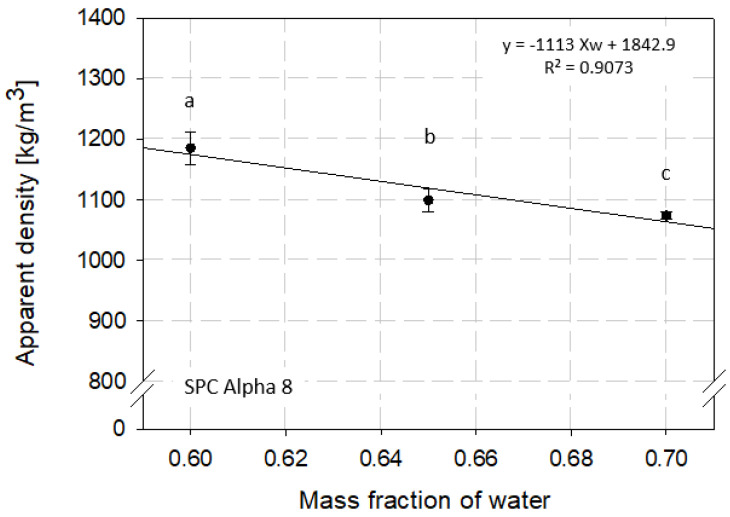
Effect of water mass fraction (moisture content) on apparent density of high-moisture extruded samples. Samples were extruded using a Werner & Pfleiderer ZSK 25 extruder. Different letters indicate grouping based on significant differences (*p* < 0.05).

Structural properties of food products are affected by various factors, such as material moisture content, material morphology, process method, and condition [6,10,34,49]. Since the texturization of high-moisture extruded samples is mainly influenced by HME process conditions such as material temperature, SME, and material pressure, these conditions could also influence the product density [33,50,51,52,53]. However, no significant density differences between different sensory-evaluated textured samples could be found (*p* > 0.05). Therefore, for each water content, a mean density was calculated (Figure 3).

In contrast to the texturization degree, the water mass fraction used in the HME process revealed an influence on apparent density. The apparent density decreased with increasing water content (Figure 3). The average density of the samples extruded at 60% water content showed a density of 1185.31 kg/m^3^ (±27.11). The samples with 65 and 70% moisture content had a density of 1098.86 kg/m^3^ (±18.58) and 1074.01 kg/m^3^ (±6.34), respectively. The decrease in density with increased water content was expected as the dry solid density is higher than the water density [34]. The density of the HMESs was significantly different for all tested water contents (*p* < 0.05).

To mathematically describe the effect of the tested moisture content on the apparent density a linear regression model was applied to the data and a regression equation of $\rho_{ap}=-1113\;X_{W}+1842.9$ with a coefficient of determination of R^2^ = 0.91 could be derived. The dependence of density on water content has already been studied and published for different food systems [44,54,55]. However, density dependence for HME products has not yet been studied. Baik and Mittal [28] published similar apparent densities in the range of 1053–1251 kg/m^3^ for tofu products with water contents between 0.34 and 0.73 (w.b.) and revealed a regression equation of $\rho_{ap}=-474\;X_{W}+1400$. These results are in good correlation to our density model-based results for HME samples.

Based on the composition of a food product, Choi and Okos [4] developed a model similar to the specific heat capacity model to predict the true density, which is the density of a pure substance or a material calculated from its components. However, the model of Choi and Okos [4] does not take into account structural effects, air phase, or interaction between phases and, hence, has limited application. Rahman and Driscoll [36] extended the Choi and Okos [4] model, including mass and volume conservation to consider interactions of phases and formation of an air phase. Therefore, a density prediction model was developed using mass and volume conservation, and new terms were introduced to account for interactions of the phases and formation of an air phase during processing. Another approach to determine the density of HMESs is via the HME process. Thus, the density of an HME sample can be predicted online based on the extrusion throughput, the cross-sectional area of the cooling die used, and the average flow rate of the product when exiting the cooling die. All prediction models mentioned are summarized in Table 7, and our experimental density data measured at a temperature of 20 °C were compared to those models. The relative errors were calculated in percent with respect to the experimental data.

Relative errors between −11.24 and 2.54% could be calculated. Despite the inclusion of the excess volume, the densities for the model of Rahman and Driscoll [36] are predominantly underestimated; the same can be observed for the original model of Choi and Okos [4]. The excess volume calculation function does not optimally reflect the fibrous structure in the high-moisture extruded samples. The density model of Baik and Mittal [28] based on tofu products also underestimates the density of high-moisture extruded samples, especially for those samples extruded at 60% water content, but overall the deviations were lower in comparison to Rahman and Driscoll’s model.

The calculation of the density via process conditions is a good opportunity to measure the density of the products inline and could be applied to adjust the process conditions if, e.g., HMMA products with a certain density are desired. However, this method includes some measurement uncertainties, as inline monitoring of the parameters, such as extrusion throughput, the cross-sectional area of the cooling die, and especially the average flow rate, must be very exact to avoid over- or underestimation of the apparent density. As shown in Table 7, the approach is feasible and gives a rough estimation but is not as exact as the derived regression model from our experimental data.

The density prediction using the regression equation derived in this study with an R^2^ of 0.90 leads to a percentage deviation of −0.95% (HMES, Xw = 0.60) to 1.87% (HMES, Xw = 0.70) from the geometric dimension method, and is negligible. Thus, the prediction of the density of high-moisture extruded samples with experimentally or theoretically derived models is possible but the origin of the models’ must be considered to avoid significant deviations. However, a definite statement on the dependency between texture and apparent density in the tested samples cannot be made due to the small number of high-moisture extruded samples investigated and this could be further investigated in future studies.

Due to the lowest relative errors of our regression model with respect to the measured data, our model should be the preferred density prediction model for HME samples. According to Rao et al. [8], accuracies of more than 2–5% are rarely required for most engineering heat transfer calculations performed in commercial food heating or cooling applications. Further, the variation in composition, size, and shape of most food products precludes the necessity for higher accuracies [8] and Rahman and Driscoll [36] stated that for the prediction of density, an error of less than 3% is normally tolerable for design and process calculations.

### 3.3. Prediction of Thermal Diffusivity

**Figure 4:**
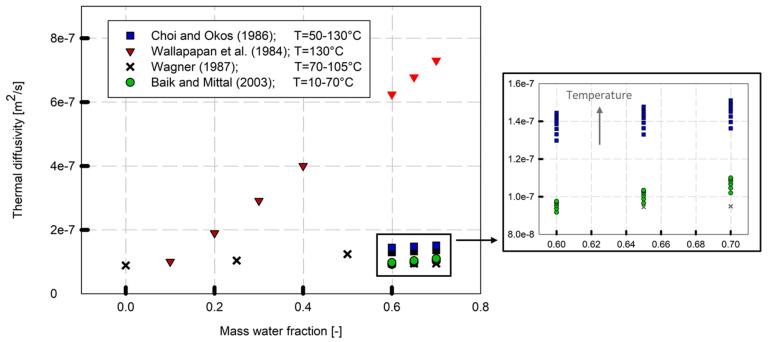
Comparison of theoretical and experimental models for predicting thermal diffusivity of high-moisture extruded samples. Data points displayed with a lighter color represent extrapolated data. Refs. [13,20,27,28].

In this study, the thermal diffusivity was predicted using theoretical and empirical models from the literature, as listed in Table 4, and a comparison of the model equations is shown in Figure 4.

The thermal diffusivity of high-moisture extruded samples was affected by the water content and increased as moisture content increased. Similar results have been calculated and published previously [13,28]. Temperature and water content are major factors affecting thermal diffusivity compared to fat, protein, and carbohydrates [7]. Food composition and porosity are further influential factors [34].

The calculation of the thermal diffusivity was based on the prediction models shown in Table 4. The validity of these models is limited by their studied parameter range. Comparing the thermal diffusivity values calculated using different theoretical and experimental models, differences become apparent. The thermal diffusivity model from Wallapapan et al. [13] showed significantly higher values in comparison to the other models. The data calculated based on the models of Choi and Okos [20] and Baik and Mittal [28] resulted in similar values. An extrapolation of Wagner’s model [27] also showed good agreement with these values. In particular, for mass water fractions of 0.60 and 0.65, the values of Wagner [27] and Baik and Mittal [28] were almost identical. For Wagner [27], however, only average thermal diffusivity values for the temperature range from 70 to 105 °C were given.

Although the thermal diffusivity values for high-moisture extruded samples were not experimentally determined in this study, the models from Choi and Okos [20] and Baik and Mittal [28] could be used as a reasonable assumption for the thermal diffusivity, since they showed similar results for the water content range of HMESs, regardless of the origin of the models. Thus, for the determination of the thermal conductivity (k), the thermal diffusivity (α) was calculated based on Choi and Okos [20] and Baik and Mittal [28]. Those models were chosen as the parameter range of Choi and Okos’ model was like ours, and Baik and Mittal’s model was based on a similar product (Table 4).

### 3.4. Prediction of Thermal Conductivity

The prediction of thermal conductivity of foods is complex and the obtained results can vary by two orders of magnitude [34]. Thermal conductivity depends on composition, structure, and temperature. The thermal conductivity of water is much higher than that of other food components (protein, fat, and carbohydrate), and therefore the water content of foods has a significant effect on the thermal conductivity of foods containing water [56]. Furthermore, the thermal conductivity is not the same for all spatial directions for non-isotropic products. The structure of fibrous materials such as meat, fish, or analogue products affects thermal conductivity [2,8,34]. The thermal conductivity parallel to fibers is 1–20% higher than perpendicular to fibers, e.g., in red meat, poultry, and fish products [2,11,12].

Thermal conductivity (k) combines thermal diffusivity (α) with density (ρ) and specific heat capacity (c_p_) (Equation (6)). The specific heat and density models developed in this work were applied to calculate the thermal conductivity. As mentioned above, the thermal diffusivity was still an unknown variable in our study and therefore it was predicted using the model equations of Baik and Mittal [28] and Choi and Okos [20] (Table 4) and could be used as a reasonable assumption of thermal diffusivity prediction for HME samples (Figure 4).

(6) $$k=\alpha \;\rho \;c_{p}$$

**Figure 5:**
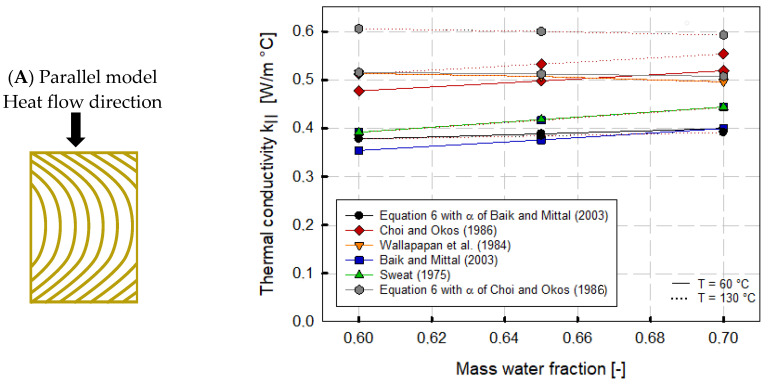

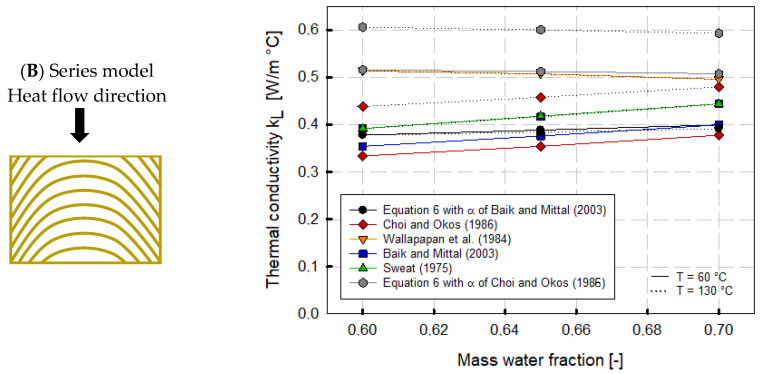
Thermal conductivity of high-moisture extruded samples as a function of mass water fraction and heat flow direction using different theoretical and experimental models. (**A**) Parallel model: Heat flow direction parallel to fibers. (**B**) Series model: Heat flow direction in series to fibers. Solid lines correspond to values at a temperature of 60 °C; dashed lines correspond to values at a temperature of 130 °C. Refs. [12,13,20,28].

To consider the multilayered fibrous structure of HMESs, the thermal conductivity was further calculated according to the serial and parallel models of Choi and Okos [20]. The calculated thermal conductivity values were compared to various theoretical and experimental models taken from the literature such as Wallapapan et al. [13], Baik and Mittal [28], and Sweat [12] (Table 5), and the comparison of the calculated thermal conductivities based on the heat flow direction is shown in Figure 5. A moisture content of 60, 65, and 70% and temperatures of 60 and 130 °C were chosen, as these temperatures covered the domain in which all literature models used were valid.

Figure 5 clearly shows that the thermal conductivity differed for the parallel (Figure 5A) and series models (Figure 5B), and a dependency existed between thermal conductivity and water content. Thus, thermal conductivity increased with increasing water content, except for the values obtained from the model of Wallapapan et al. [13]. Here, a slight decrease in thermal conductivity at a high mass water fraction (Xw = 0.60–0.70) could be observed. As this model is valid for the water content of 9.2 to 39.1% (w.b.), the decrease could be due to the extrapolation to a water content of 70%.

Furthermore, the various models depicted different temperature dependencies. The models of Sweat [12] based on meat and fish products and the model of Wallapapan et al. [13] based on defatted soy flour were not temperature sensitive neither for the parallel nor the series model (Table 5 and Figure 5A,B). The thermal conductivity calculated using Equation (6) with the calculated thermal diffusivity of Baik and Mittal [28] showed negligible temperature dependency, which could be attributed to the low sensitivity of the temperature term of the thermal diffusivity used (Figure 4). In contrast, the thermal conductivity calculated using Equation (6) with the calculated thermal diffusivity of Choi and Okos [20] showed a clear temperature dependency.

In the parallel model, the prediction models of Choi and Okos [20] and Baik and Mittal [28] depicted similar temperature sensitivities, with higher thermal conductivity values for Choi and Okos’ model (Figure 5A). Thus, values of 0.477 W/m °C at 60 °C and 60% moisture and 0.519 W/m °C at 60 °C and 70% moisture could be calculated. At 60 °C, the thermal conductivity based on the regression equation of Baik and Mittal [28] ranged from 0.354 to 0.400 W/m °C at 60% moisture and 70% moisture, respectively. The temperature dependence of Choi and Okos’ model [20] is enhanced in the serial model, where the heat flow is perpendicular to the multilayered fibrous structure.

Hence, differences become apparent when comparing the parallel and serial models. For the serial model (Figure 5A), the thermal conductivities calculated by Choi and Okos [20] were close to the models of Sweat [12] for meat and fish products and Baik and Mittal [28] for tofu. Both products have a defined texture, although tofu has a spongy texture rather than a fibrous texture compared to HMMA products. Baik and Mittal [28] were able to find good comparability of experimental results with the serial model in their studies. The models of Wallapapan et al. [13] and Sweat [12] were structure independent, and the thermal conductivity calculated via the mathematical relationship (Equation (6)) between density, specific heat, and thermal diffusivity did not include any directional heat flow dependence.

Baik and Mittal’s thermal diffusivity equation is based on experimental data on tofu, and Choi and Okos’ thermal diffusivity model is based on experimental data on various water-containing foods. Figure 5 depicts a significantly higher thermal conductivity using the prediction equation for thermal diffusivity of Choi and Okos [20] than the prediction model of Baik and Mittal [28]. Calculating the thermal conductivity via Equation (6) with α derived from Choi and Okos [20] using our measured c_p_ and apparent density values, the thermal conductivity was 0.54 W/m °C at Xw = 0.60 and T = 60 °C.

In comparison, a thermal conductivity value of 0.36 W/m °C could be determined at Xw = 0.60 and T = 60 °C for the thermal conductivity prediction with Equation (6) when α was calculated via Baik and Mittal [28].

The comparison of different models for predicting the thermal conductivity of HMESs leads to the conclusion that its thermal conductivity could be calculated using the model of Baik and Mittal [28] or Equation (6) with our generated data and the thermal diffusivity from Baik and Mittal [28]. The model of Baik and Mittal [28] should be chosen over the other literature models, as this model is based on tofu and thus closer to HME products than the model of Choi and Okos [20], which is based on various high-moisture food products. Moreover, unlike the models of Wallapapan et al. [13] and Sweat [12], this model is temperature dependent.

The model from Baik and Mittal could certainly be used to calculate the thermal conductivity of HME products, however, experimentally derived models, like our model, which is based on experimental data, should be prioritized because of their higher accuracy. Therefore, further research, such as simulation of the HME process, should be conducted using our model, as it is closest to the product to be studied.

## 4. Conclusions

The aim of this study was to determine data-driven prediction models for the thermophysical properties, specific heat capacity (c_p_), and apparent density (ρ) of high-moisture extruded samples with different sensory-evaluated textures and water contents and to mathematically describe their thermophysical properties based on experimental data. The derived models were compared to non-HME-based literature models that were based: (i) on food constituents and thus cover a broad range of food products, (ii) on soy protein ingredients, or (iii) on meat or fish products, which are supposed to be mimicked by HME [4,12,13,20,23,27,28,34]. Currently, there are no models in the literature that mathematically describe thermophysical properties such as specific heat capacity, apparent density, thermal diffusivity, and thermal conductivity for high-moisture extruded products. Therefore, one breakthrough established in this study was the implementation of multilinear regression models describing the thermal properties specific heat capacity and apparent density for HME samples. The thermal diffusivity (α) and the thermal conductivity (k) were calculated based on literature models and generic equations (Equation (6)), including our data-driven models for c_p_ and the apparent density.

It can be concluded that the models from Baik and Mittal [28], due to their product-related similarity (based on tofu), led to similar results in comparison to our models. Thus, using experimentally and data-driven models which are process or product dependent has the advantage of much higher accuracy in comparison to, e.g., theoretical food models or generic models.

The results of our study demonstrate the applicability of simple models, utilizing experimental data, for estimating thermophysical properties of HMESs. It is important to note that the validity of these models is limited to the narrow range of tested water content. However, considering the significance of the 60–70% water content domain in the HME process, these simple models hold potential for optimizing HME operations. Consequently, further investigations are warranted to reinforce and expand upon these promising findings. Additionally, these findings could help to understand the texturization effect during HME in the future and to produce HME samples with defined textures by implementing the thermophysical property models derived within this study in numerical simulations to investigate the texturization and structure formation effect in the cooling die.

Similar approaches using thermophysical properties of plant-based proteins to evaluate and predict their behavior during HME are detailed and discussed in Högg and Rauh [30]. In the mentioned study, the focus is on untreated plant-based proteins that were not high-moisture extruded.

## Figures and Tables

**Table 1:** Composition of the soy protein concentrate SPC Alpha 8 based on the supplier’s specification (Solae 2015).

	Unit/Legend	SPC Alpha 8
**Composition**		
**Dry matter (TS)**	g/100 g raw material	95.4
	Protein	66.5
	Fat	1.6
	Ash	6.8
	Carbohydrates (incl. 18 g/100 g dietary fibers)	20.5

**Table 2:** High-moisture extruded samples for characterization of thermophysical properties. HME samples were selected based on extruded water content and evaluated sensory texture. For further details, refer to Högg and Rauh [30].

Experimental Plan					Responses			
	Process Variables				Process Response			Product Response
No.	Moisture content [%]	Screw speed [rpm]	Mass flow rate [kg/h]	Barrel temperature [°C]	Material temperature [°C]	Extruder pressure [bar]	SME [Wh/kg]	Texture
1	60	400	13	160	133.10	21.06	30.68	well-textured
2	60	400	13	120	104.85	16.73	26.20	poorly textured
3	60	400	13	140	116.4	15.07	20.81	textured
4	65	310	9.5	170	137.76	9.69	9.71	textured
5	70	400	13	160	120.11	6.63	6.09	textured

**Table 3:** Criteria for classifying HME samples as well-textured, textured, and poorly textured, as determined by sensorial analyses.

Cluster	Description and Example of Texture
Poorly textured	Soft, mushy, and brittle HME samples displaying no multilayered, fibrous structure, with a structure like shortcrust pastry.
	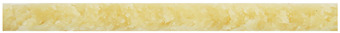
Textured	HME samples displaying a slightly fiber-like structure, a weak parabolic pattern, when manually torn apart, as well as the trend of a V-shape pattern, if cut longitudinally to the flow direction.
	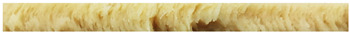
Well-textured (=HMMA)	HME samples displaying firm and defined multilayered, fibrous structures, a pronounced parabolic pattern, and a well-defined V-shape pattern.
	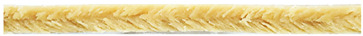

**Table 4:** Mathematical and empirical models from literature to predict thermal diffusivity.

Food Product	Temperature	Prediction Model *		Reference
Various high-moisture food products	T=−40–150 °C	$\alpha =\overset{n}{\underset{i=1}{\sum }}E_{i}\;\alpha_{i}$ with $E_{i}=\frac{\frac{M_{i}}{\rho_{i}}}{\sum_{i=1}^{n}\frac{M_{i}}{\rho_{i}}}$		[20]
		Water	$\alpha_{Water}=1.3168\times 10^{-7}+6.2477\times 10^{-10}\;T\;-2.4022\times 10^{-12}\;T^{2}$	
		Protein	$\alpha_{Protein}=6.8714\times 10^{-8}+4.7578\times 10^{-10}\;T\;-1.4646\times 10^{-12}\;T^{2}$	
		Fat	$\alpha_{Fat}=9.8777\times 10^{-8}-1.2569\times 10^{-10}\;T\;-3.8286\times 10^{-14}\;T^{2}$	
		Carbohydrate	$\alpha_{CHO}=8.0842\times 10^{-8}+5.3052\times 10^{-10}\;T\;-2.3218\times 10^{-12}\;T^{2}$	
		Fiber	$\alpha_{Fiber}=7.3976\times 10^{-8}+5.1902\times 10^{-10}\;T\;-2.2202\times 10^{-12}\;T^{2}$	
		Ash	$\alpha_{Ash}=1.2461\times 10^{-7}+3.7321\times 10^{-10}\;T\;-1.2244\times 10^{-12}\;T^{2}$	
Defatted soy flour with Xw = 0.09–0.39	T=130 °C	$\alpha =2.76\times 10^{-8}+35.74\times 10^{-8}\;X_{w}\;-14.90\times 10^{-11}\;X_{w}\;\rho +65.56\times 10^{-11}\;\left(X_{w}{}^{2}\;\rho \right)$		[13]
Soy dough with Xw = 0–0.50	T=70–105 °C	Xw = 0: $\alpha =8.89\;10^{-8}$ Xw = 0.25: $\alpha =10.37\;10^{-8}$ Xw = 0.5: $\alpha =12.43\;10^{-8}$		[27]
Tofu with Xw = 0.34–0.76 (w.b.)	T=6–74 °C	$\alpha =\left(0.0816-0.05682\;X_{w}+0.1164\;X_{w}{}^{2}+6.866\times 10^{-4}\;X_{w}{}^{2}\;T-5.17\times 10^{-6}X_{w}{}^{2}\;T^{2}\right)\times 10^{-6}$		[28]

* Thermal diffusivity in m^2^/s; calculation of ρ according to the regression equation established in this study (more information see Last Table, pages 12 and 13).

**Table 5:** Theoretical or experimental models for predicting the thermal conductivity of high-moisture extruded samples.

Food Product	Temperature	Prediction Model		Reference
Various high-moisture food products	T=−40–150 °C	$k_{\|}=\overset{n}{\underset{i=1}{\sum }}E_{i}\;k_{i}\;;\;\frac{1}{k_{⊥}}=\overset{n}{\underset{i=1}{\sum }}\frac{E_{i}}{k_{i}}$		[20]
		Water	$k_{W}=5.7109\times 10^{-1}+1.7625\times 10^{-3}\;T\;-6.7036\times 10^{-6}\;T^{2}$	
		Protein	$k_{P}=1.7881\times 10^{-1}+1.1958*10^{-3}\;T\;-2.7178*10^{-6}\;T^{2}$	
		Fat	$k_{Fa}=1.8071\times 10^{-1}-2.7604*10^{-3}\;T\;-1.7749\times 10^{-7}\;T^{2}$	
		Carbohydrate	$k_{CHO}=2.0141\times 10^{-1}+1.3874\times 10^{-3}\;T\;-4.3312\times 10^{-6}\;T^{2}$	
		Fiber	$k_{Fi}=1.8331\times 10^{-1}+1.2497\times 10^{-3}\;T\;-3.1683\times 10^{-6}\;T^{2}$	
		Ash	$k_{Ash}=3.2962\times 10^{-1}+1.4011\times 10^{-3}\;T\;-2.9069\times 10^{-6}\;T^{2}$	
Defatted soy flour with Xw = 0.09–0.39	T=130 °C	$k=-0.228+0.249\times 10^{-3}\;\rho +1.304\;X_{w}-0.926\;X_{w}{}^{2}$		[13]
Tofu with Xw = 0.34–0.72 (w.b.)	T=6–74 °C	$k=0.2112+0.0008943\;X_{w}\;T+0.3077\;X_{w}{}^{2}$		[28]
Meat and fish products with Xw = 0.60–0.80 (w.b.)	T=0–60 °C	$k=0.08+0.52\;X_{w}\;$		[12]
HME sample with Xw = 0.60–0.70 (d.b.)		$k=\alpha \;\rho \;c_{p}$ Specific heat $c_{p}$ and density ρ were calculated with models developed in this study, α was taken from [28] (2003) and [9] (1986), respectively		Mathematical calculation

**Table 6:** Theoretical or empirical methods from literature for predicting the specific heat capacity of food. The percentage deviation describes the difference between the experimentally obtained value vs. the value calculated by the respective model.

Food Product	Temperature	Prediction Model		Percentage Deviation (at T = 90 °C and Xw = 0.60) †	Reference
Broad range of food products Average for all types of carbohydrates ($X_{C}$), protein ($X_{P}$), fat ($X_{F}$), and ash ($X_{A}$)	−40 °C<T<150 °C	$c_{p}=\overset{n}{\underset{i=1}{\sum }}c_{pi}X_{i}$		HME sample: 0.84%	[4]
		Water	$c_{pW}=4176.2-0.0909\;T+5.4731\times 10^{-3}\;T^{2}$		
		Protein	$c_{pP}=2008.2+1.2089\;T-1.3129\times 10^{-3}\;T^{2}$		
		Fat	$c_{pF}=1984.2+1.4733\;T-4.8008\times 10^{-3}\;T^{2}$		
		Carbohydrate	$c_{pC}=1548.8+1.9625\;T-5.9399\times 10^{-3}\;T^{2}$		
		Fiber	$c_{pFi}=1845.9+1.8306\;T-4.6509\times 10^{-3}\;T^{2}$		
		Ash	$c_{pA}=1092.6+1.8896\;T-3.6817\times 10^{-3}\;T^{2}$		
Defatted soy flour at MC of 9.2 to 39%	T=130 °C (DSC method)	$c_{p}=1749+3363\;X_{W}$ (c_p_ in J/kg °C)		HME sample: 5.54% at T = 130 °C and Xw = 0.60	[13]
Defatted soy dough at MC of 0 to 70%	25 °C<T<170 °C (DSC method)	50 % MC and T = 90 °C: $c_{p}=3055.12\;J/\mathrm{kg}\;K$		HME sample: −7.23% at T = 90 °C and Xw = 0.50	[27]
Soy dough	T=20 °C	$c_{p}=1480+2170\;X_{W}$ (c_p_ in J/kg °C)		HME sample: −1.10% at T = 20°C and Xw = 0.60	[40]
Tofu Xw = 0.3–0.7 (w.b.)	10 °C<T<105 °C (modulated DSC method)	$c_{p}=2055+1064\;X_{W}+1596\;X_{W}{}^{2}+1.06\;T$ ($R^{2}=0.959$) (c_p_ in J/kg °C, T in °C)		HME sample: −1.46%	[28]
Turkey Xw = 74.88 %	34 °C<T<82 °C (modulated DSC method)	$c_{p}=3003.3+4.567\;T\;$ ($R^{2}=0.998$) (c_p_ in J/kg °C, T in °C)		HME sample: −5.19% at T = 80 °C and Xw = 0.7488	[18]
**Regression model** Xw = 0.6–0.7 (d.b.)	40 °C<T<115 °C (DSC method)	$c_{p}=2341.64+1201.57\;X_{W}+3.8958\;T$ $(R^{2}=0.96$)		HME sample: −0.29% ‡	**Developed in this study**

† Unless otherwise stated, equation of the multilinear regression model was used. ‡ Compared to value measured c_p_ at 90 °C (3423 J/kgK) via µDSC.

**Table 7:** Theoretical or experimental models for predicting the density of high-moisture extruded samples. The percentage deviation describes the difference between the experimentally obtained value vs. the value calculated by the respective model.

Food Product	Temperature	Prediction Model		Percentage Deviation (at T = 20 °C) †	Reference
Fresh seafood Xw = 0.73–0.86 (w.b.)	T=20±3 °C	$\rho_{ap}=\left(1-\varepsilon_{ex}\right)\frac{1}{\sum_{i=1}^{n}\frac{X_{i}}{\rho_{i}}}$		HME sample, Xw = 0.60: −11.24% HME sample, Xw = 0.65: −4.83% HME sample, Xw = 0.70: −3.20%	[36] based on [4]
		Water	$\rho_{W}=997.18+3.1439\times 10^{-3}\;T-3.7574\times 10^{-3}\;T^{2}$		
		Protein	$\rho_{P}=1329.9-5.1840\times 10^{-1}\;T$		
		Fat	$\rho_{F}=\;\;925.6-4.1757\times 10^{-1}\;T$		
		Carbohydrate	$\rho_{C}=1599.1-3.1046\times 10^{-1}\;T$		
		Fiber	$\rho_{Fi}=1311.5-3.6589\times 10^{-1}\;T$		
		Ash	$\rho_{A}=2423.8-2.8063\times 10^{-1}\;T$		
		Excess volume	$-\varepsilon_{ex}=\left(93.24\;X_{w}+42.97\;X_{P}+2460\;X_{F}-4256\;X_{A}-1086\;X_{C}\right)\times 10^{-4}$		
Tofu Xw = 0.34–0.73 (w.b.)	6 °C<T<74 °C	$\rho_{ap}=-474\;X_{W}+1400$ ($R^{2}=0.961$)		HME sample, Xw = 0.60: −5.88% HME sample, Xw = 0.65: −0.63% HME sample, Xw = 0.70: −0.54%	[28]
HME sample Xw based on HME process	T Based on HME process	$\rho_{ap}=\frac{\dot{m}}{\dot{V}}=\frac{\dot{m}}{v\times A_{▭}}$		HME sample, Xw = 0.60: −2.34% HME sample, Xw = 0.65: −3.32% HME sample, Xw = 0.70: −2.71%	HME process (based on process conditions)
HME sample Xw = 0.60–0.70 (d.b.)	T=20±5 °C	$\rho_{ap}=-1113\;X_{W}+1842.9$ ($R^{2}=0.9073$)		HME sample, Xw = 0.60: −0.86% HME sample, Xw = 0.65: 1.87% HME sample, Xw = 0.70: −0.95%	Regression equation (experimental data)

With density $\rho \;\mathrm{in}\;\mathrm{kg}/m^{3}$; average flow rate v in m/s; throughput $\dot{m}\;\mathrm{in}\;\mathrm{kg}/s$; cross-sectional area of the cooling die $A_{▭}\;{\mathrm{in}\;m}^{3}$; † unless otherwise stated, all relevant process-related data can be found in Table 2.

## Data Availability

The data presented in this study are available on request from the corresponding author.
